# Fuzzy Logic Controlled Simulation in Regulating Thermal Comfort and Indoor Air Quality Using a Vehicle Heating, Ventilation, and Air-Conditioning System

**DOI:** 10.3390/s23031395

**Published:** 2023-01-26

**Authors:** Konguvel Rajeswari Subramaniam, Chi-Tsun Cheng, Toh Yen Pang

**Affiliations:** School of Engineering, STEM College, RMIT University, 124 La Trobe St, Melbourne, VIC 3000, Australia

**Keywords:** heating, ventilation, air-conditioning system, thermal comfort, predicted mean vote, indoor air quality, carbon dioxide, fuzzy logic control

## Abstract

Conventional heating ventilation and air-conditioning (HVAC) controllers have been designed to mainly control the temperature of a confined compartment, such as a room or a cabin of a vehicle. Other important parameters related to the thermal comfort and indoor air quality (IAQ) of the confined compartment have often been ignored. In this project, IAQ in the vehicle cabin was represented by its carbon dioxide (CO_2_) concentration, and the occupants’ thermal comfort levels were estimated with the predicted mean vote (PMV) index. A new fuzzy logic controller (FLC) was designed and developed using the MATLAB fuzzy logic toolbox and Simulink to provide a nonlinear mapping between the measured values, i.e., PMV, temperature, CO_2_, and control parameters (recirculation flaps, blower’s speed, and refrigerant mass flow rate) of a vehicle HVAC system. The new FLC aimed to regulate both in-cabin PMV and CO_2_ values without significantly increasing overall energy consumption. To evaluate the effectiveness of the proposed FLC, a cabin simulator was used to mimic the effects of different HVAC variables and indoor/outdoor environmental settings, which represented the in-cabin PMV and IAQ readings. Results demonstrated that the new FLC was effective in regulating the in-cabin PMV level and CO_2_ concentration, at desirable levels, by adaptively controlling the opening and closing of the recirculation flap based on in-cabin temperature and CO_2_ readings, while maintaining an average-to-good energy consumption level. The proposed FLC could be applied to a large variety of HVAC systems by utilizing low-cost sensors, without the need to significantly modify the internal design of the HVAC system.

## 1. Introduction

While it is expected that 9 out of 10 new cars sold worldwide in 2030 will be electric vehicles, internal combustion engine vehicles will still dominate the road for a few more decades. Regardless of the type of vehicle, the mechanisms of their heating, ventilation, and air-conditioning (HVAC) systems are very similar [1]. In general, the HVAC systems in any vehicle serve to maintain ventilation and the health of the occupants in terms of thermal comfort and vehicle cabin indoor air quality (IAQ). In a conventional HVAC system, the occupant’s preferred thermal comfort and IAQ are normally measured in terms of temperature and humidity. Thermal comfort is a subjective expression of an occupant’s thermal sensation that is estimated from objective measurements collected from the surrounding environment. Besides temperature and humidity, thermal comfort can further include two other environmental factors, namely, mean radiant temperature and air velocity, and two human factors, namely, clothing insulation (clo) and metabolic equivalent (MET) rate, as mentioned in ASHRAE 55-2021 [2]. This paper aimed to design a vehicle HVAC controller for regulating the thermal comfort of occupants and in-cabin IAQ at desirable levels without causing a significant increase in energy consumption or requiring sophisticated modifications to the HVAC system.

### 1.1. Background Information

#### 1.1.1. PMV and IAQ

Thermal comfort can be estimated using a steady-state model or an adaptive model as proposed in [3] and [4], respectively. In the 1970s, Franger [3] developed a steady-state model to estimate a person’s thermal comfort level, also widely known as the predicted mean vote (PMV) index model, which estimated the mean thermal sensation of a group of people in an indoor space based on the environmental factors and human factors mentioned above. PMV has been widely accepted by international standards, such as ASHRAE 55-2021, ISO-7730, EN-15251, CEN CR 1752, to quantify the levels of human thermal comfort in a closed air-conditioned space.

Apart from thermal comfort, the cabin IAQ of a vehicle, in particular the carbon dioxide (CO_2_) concentration, can have a significant impact on the health and safety of its driver and passengers. The increase in CO_2_ concentration in the vehicle cabin can cause drowsiness to the driver, which might lead to fatal accidents. Under most circumstances, the outdoor air contains around 400 parts per million (ppm) of CO_2_. Inside the cabin, CO_2_ is continuously generated by passengers due to their respiration and metabolism. As the vehicle compartment is relatively small concerning the number of occupants in it, CO_2_ can be accumulated quickly, especially when the recirculation mode of the vehicle HVAC system is enforced [5]. To avoid health hazards to its occupants, it is suggested that the CO_2_ concentration for a closed compartment should not exceed 1000 ppm [6,7]. Besides CO_2_ concentration, the IAQ inside a vehicle cabin can also be determined by two major pollutants, namely, particulate matter (PM) and volatile organic compounds (VOCs) [5,8,9]. The HVAC filters can remove most PM and other IAQ pollutants through diffusion, interception, and impaction. The amount of VOCs inside the cabin is largely dependent on the vehicle’s interior arrangements. It is stated in [9] that HVAC filters are not effective to filter VOCs. However, VOCs, such as CO_2_ and other gases, can be regulated by opening the recirculation flap and bringing fresh air in [5,9]. In conventional vehicle HVAC systems, users can manually open the recirculation flap to allow fresh air to flow into the cabin. When the indoor and outdoor temperature and humidity are significantly different, most users may choose to partially or completely close the recirculation flap to yield faster cooling/heating. On the one hand, such practices can improve the efficiency of the HVAC system in cooling/heating the cabin as air is being recirculated. On the other hand, such an action forms a closed loop in the HVAC system which causes CO_2_ concentration to increase. Zhao et al. [10] stated that in electrical vehicles, to reduce energy consumption and prolong milage, the recirculating air percentage can be even higher than that of internal combustion engine vehicles. All these configurations and settings, if not accompanied by proper monitoring and control mechanisms, can cause the in-cabin CO_2_ concentration to go beyond the safety limit. Therefore, apart from thermal comfort, HVAC systems of vehicles should continuously regulate the in-cabin CO_2_ level to achieve better IAQ. There are limited studies on the usage of quantifiable thermal comfort index (e.g., PMV and IAQ) as dual input parameters in HVAC system control and design. There are also limited studies on the usage of quantifiable thermal comfort index (e.g., PMV) and IAQ as dual input parameters in HVAC system control and in regulating in-cabin PMV and CO_2_ values at some predefined thresholds without causing a significant increase in the overall energy consumption or requiring major hardware modifications.

#### 1.1.2. Vehicle HVAC System

**Figure 1:**
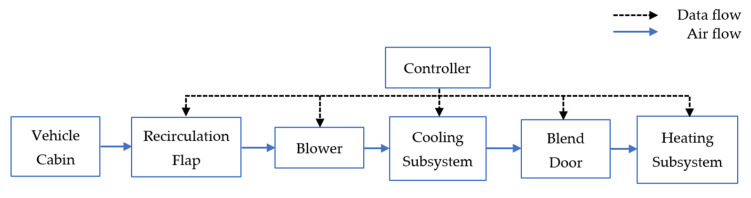
An overview of the cooling and heating subsystems in a conventional vehicle HVAC system.

A typical vehicle HVAC system (Figure 1) normally comprises cooling and heating subsystems, a blower, and filter components. The main component of the cooling subsystem is the refrigeration cycle loop, which contains a compressor, condenser, thermostatic expansion valve, and evaporator.

As illustrated in Figure 1, the blower, which is controlled by a controller, draws the cabin air or the fresh air from the outdoor environment into the HVAC system for cooling or heating. The recirculation flap controlled by the controller regulates the amount of fresh air entering the vehicle cabin through the HVAC system. The mixture of the cabin air and the fresh air then passes through the cooling subsystem, which reduces their temperatures, and then reaches the blend door which is also known as the temperature control flap. When heating is required, the blend door adjusts the flap such that a portion of the cold air can pass through the heating component. In internal combustion engine vehicles, the heating subsystem has two components, namely, (i) the radiator, which gains heat from the engine, and (ii) the positive temperature coefficient heater (i.e., an electric heater). The process is considered as efficient as when heating is required, the air can be heated up by the waste heat from the engine. Under extremely cold weather, only when the engine is not hot enough to heat the air, the positive temperature coefficient heater will need to be turned on to heat the cold air to the desired temperature level [11].

#### 1.1.3. Fuzzy Logic Controller

**Figure 2:**
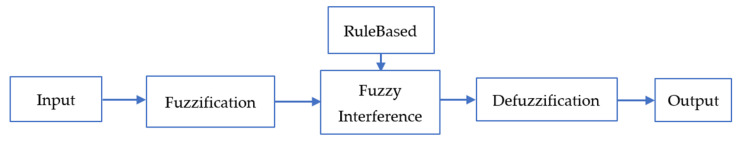
Overview of a fuzzy logic-based controller.

A fuzzy logic controller (FLC) has been demonstrated as a good candidate for system control, which imitates the human decision-making processes, i.e., range-to-point or range-to-range control [12]. FLC has relatively low computational complexities for mapping input and output parameters of systems with non-binary natures and non-linear relationships. It is often applied for mapping non-linear input data captured from sensors to desirable control parameters for system components such as actuators. An overview of a typical fuzzy logic-based controller is shown in Figure 2. Fuzzification is used to assign numerical data to fuzzy sets. Fuzzy sets consist of elements that represent the degrees of their membership (i.e., the importance or severity of a reading). In general, the degrees of membership of the fuzzy sets lie between 0 and 1, which are expressed as membership functions. The fuzzy inference then generates outputs from the fuzzified data based on sets of pre-defined rules. Finally, defuzzification maps the outputs of the fuzzy inference process back into their numerical forms. Details on the operation principles of a generic fuzzy controller can be found in [12].

Once the system has been set up, the whole fuzzy logic inferencing process will only involve simple arithmetic operations, which makes it highly desirable for real-time control applications and to be implemented in low-cost low-power microcontrollers. While the majority of existing fuzzy controllers for HVAC systems have been designed for reducing energy consumption, adaptive intelligent controller structures and algorithms that incorporate human thermal comfort are still lacking.

### 1.2. Literature Review

Over the years, vehicle HVAC system control strategies have evolved from rigid rule-based switch control mechanisms to intelligent and adaptive control algorithms such as neural networks, fuzzy logic, reinforcement learning, etc. [13]. Table 1 presents the current HVAC controllers that attempted to regulate the thermal comfort and IAQ inside a vehicle cabin in terms of temperature and relative humidity in an energy-efficient manner. In [14], it has been reported that an HVAC system with a rigid rule-based control strategy, i.e.,on–off switching based on predefined thresholds, is inefficient in regulating non-linear and conflicting parameters such as in-cabin PMV and CO_2_ levels. Conventional control mechanisms, such as proportional integral derivative (PID) control, are also not suitable for applications with non-linear external environmental parameters and they can lead to unsatisfactory results in maintaining thermal comfort and IAQ inside the vehicle cabin simultaneously [15]. Behrooz et al. [14] stated that most conventional HVAC systems are equipped with proportional-integral (PI)/proportional-integral-derivative (PID) controllers; however, in some scenarios, more than one controller is required to achieve some non-trivial objectives, and can, consequently, significantly increase the complexity and cost of the implementation [16]. In [17], simulated results showed that an FLC is superior to different PID controllers in terms of response time, performance, and preserving stability. Furthermore, the FLC has reduced overshoot and steady-state error when compared to PID controllers [17]. Nevertheless, FLCs enhanced with machine learning techniques, such as machine learning-based automated fuzzy-set fine-tuning, can deliver even more promising results [14].

Table 1 presents the thermal comfort and IAQ parameters considered in existing works on vehicle HVAC system controllers designed in the past five years. Of these, only a few have considered the PMV index and IAQ simultaneously when designing vehicle HVAC system controllers, while the remaining focused on either thermal comfort or cabin IAQ. Among them, studies that involved air velocity and relative humidity often considered them as constants throughout the simulated or tested period. Moreover, approaches based on machine learning algorithms used in-cabin temperature, as the primary factor, to predict the PMV index of the occupants and, thus, regulated their thermal comfort levels without incorporating other factors. In the literature, only a few of the previous works have considered regulating CO_2_ concentration as one of their design objectives. Interestingly, when other IAQ parameters, such as PM and VOCs, have been considered as the parameters to be regulated instead of CO_2_, the corresponding controller would tend to enforce recirculation. This is because recirculation will increase the chance for PM and VOCs to be captured by the filter in the HVAC system. However, such a design can be catastrophic when regulation of CO_2_ concentration has become one of the design criteria. Among all the works presented in Table 1, only Wei et al. [5] considered all three IAQ parameters. However, their controller did not control the recirculation flap and, thus, the level of air recirculation in a compartment.

In addition to the limitations presented in Table 1, parameters such as the mean radiant temperature, human metabolic rate, and clothing factors, which are required to calculate the PMV indices, have often been assumed to be constants, as follows:

1. The mean radiant temperature was assumed to be equal to the cabin temperature;
2. The occupants could talk, sit, type, read, etc., where the average metabolic rate (MET) was approximated to be between 1.2 to 1.4 MET;
3. The clothing thermal insulation (clo) was set as less than 1.5 clo as sweaters, suit jackets, and vests were considered not to be worn.

From the literature, regulating occupants’ thermal comfort and IAQ inside the cabin in an energy-efficient manner has not been reported. In this paper, we, therefore, custom-designed a smart FLC that could regulate the estimated PMV index of the occupants and the in-cabin CO_2_ concentration by performing a sensible trade-off in terms of energy consumption of the HVAC system. Model development, evaluation, and tuning of the controller were conducted using MATLAB Simulink 2022a [24]. On the one hand, to regulate the occupants’ desirable thermal comfort, it is essential to control the air velocity and temperature. This can be achieved by controlling the blower, refrigerant flow (cooling), and hot coolant flow (heating). To regulate the cabin IAQ, fresh air from the outside environment needs to be circulated into the vehicle cabin. This can be achieved by controlling the opening and closing of the recirculation flap. The results obtained were analyzed and discussed for further improvement of intelligent HVAC controllers.

## 2. Materials and Methods

**Figure 3:**
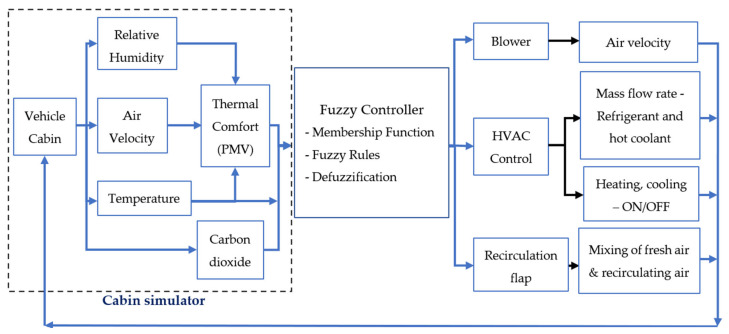
An overview of the simulator and controller subsystems in a vehicle HVAC system.

In this paper, the new FLC was designed to control the blower, heating, and cooling of the HVAC system, the mass flow rate of the refrigerant and hot coolant, and the opening and closing of the recirculation flap in the vehicle HVAC system simultaneously, as shown in Figure 3. The HVAC controller was used to regulate and meet some predefined criteria based on readings and measurements that represented the intrinsic and extrinsic factors of the cabin and its occupant(s).

### 2.1. Vehicle HVAC System and Cabin Simulator

Simulink (2022a) was used to model and simulate the vehicle cabin and its HVAC system. The vehicle HVAC system model was developed based on [25,26]. As illustrated in Figure 3, the cabin simulator continuously returned in-cabin environmental variables, namely, PMV index, cabin temperature, air velocity, and CO_2_ concentration under the inference of different simulated outdoor environmental conditions. Using the parameters obtained from the cabin simulator, the FLC then controlled the blower speed, refrigerant mass flow rate, on/off control of the heating and cooling elements, and the opening and closing of the recirculation flap of the simulated vehicle HVAC system, which was connected to the simulator, to regulate the thermal comfort and IAQ inside the vehicle cabin.

#### 2.1.1. PMV Model

The latest PMV model in ASHRAE Standard 55 [2] was adopted. The PMV model predicted the mean thermal sensation of a group of people in a closed compartment according to (i) two human factors, namely, clothing insulation and metabolic rate, which were based on some typical values obtained from the ASHRAE Standard 55, Tables 5.1 to 5.4 [2]; and (ii) four environmental factors, namely, cabin air temperature, mean radiant temperature, air velocity, and relative humidity, which were obtained from the cabin simulator.

#### 2.1.2. Temperature and CO_2_ Models

The temperature model used in the simulator was designed to dynamically update the cabin air temperature based on the heat exchange between the simulated cabin and the environment, the number of occupants in the cabin, and the CO_2_ concentration and moisture content generated by the occupants. The heat exchange process was simulated with convective and conductive heat transfer models.

**Figure 4:**
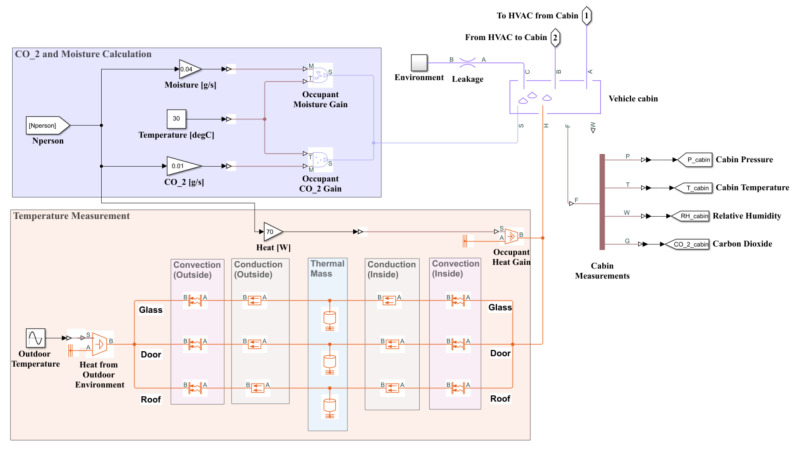
Simulink model for temperature and CO_2_ calculation.

Each occupant was assumed to generate 0.04 g/s of water vapor throughout the simulation period. The outside environment air was assumed to have a CO_2_ concentration of 400 ppm [27]. The CO_2_ concentration generated by the human body was a function of oxygen consumption level, respiratory system, and human metabolic rate [28,29]. The CO_2_ exhaled by a passenger inside the vehicle cabin was assumed to be 0.01 g/s, as that in [28,29]. An overview of the temperature and CO_2_ models implemented in MATLAB Simulink is shown in Figure 4.

### 2.2. Fuzzy Control Architecture

The proposed FLC was designed to regulate the thermal comfort, in terms of PMV index, and allowed the PMV to fluctuate between −1 and +1, the cabin indoor temperature to be regulated between 21 and 25 °C, and CO_2_ concentration to be maintained below 650 ppm without significantly increasing the overall energy consumption of the HVAC system.

#### 2.2.1. Input and Output Variables

In the simulation model, PMV, CO_2_, and cabin temperature were the input variables (Table 2) being fed into the FLC. These input variables were specified as multiple membership functions, where each input was mapped to a value between 0 and 1. These membership functions quantified the degree or level of the input elements in the fuzzy set.

The output variables of the FLC were mapped to the control variables of the HVAC system (Table 2), namely, the blower velocity, the mass flow rate of the refrigerant (compressor control) and hot coolant, and the recirculation flap. The output variables were specified from 0 (representing the component was turned off or operating at the minimum level) to 1 (indicating the fully turned on or operating at the highest level).

#### 2.2.2. Linguistic Variables

In fuzzy logic, the degree or level of a variable was first mapped to a linguistic variable. The input and output linguistic variables are shown in Table 3 and Table 4, respectively.

#### 2.2.3. Membership Functions

The membership functions are technically the mathematical functions that mapped the linguistic variables into membership degrees with values between 0 and 1 to represent their level of importance or severity. The ranges of the linguistic variables were specified based on the threshold limits specified in Table 4.

In this paper, triangular and trapezoidal membership functions were used in the fuzzy controller. The shape of a triangular membership function was defined by its lower limit (*l*), an upper limit (*u*), and a value (*m*) which represented where the function peaks out, thus *l* < *m* < *u*. Similarly, for the trapezoidal membership function, its shape was defined by its lower limit (*l*), the upper limit (*u*), a lower support limit (*l*_s_), and an upper support limit (*u*_s_) defined as *l* < *l*_s_ < *u* <*u*_s_. The triangular and trapezoidal membership functions were chosen for their low computational complexities and ease of implementation [31,32]. The trapezoidal membership functions were used to represent the extreme threshold ranges at two ends, while triangular membership functions were used to represent threshold ranges in between.

Table 4 has been deduced from Table 2 and Table 3. In Table 4, according to the CO_2_ concentration accumulated inside the cabin, the opening and closing of the recirculation flap will be executed. When the FLC executes as Low, the recirculation flap will be opened by 25%. Similarly, when it is High, the recirculation flap will be fully open. For the mass flow rate of the refrigerant and coolant, since the total mass flow rate is specified in the evaporator, when the FLC executes a linguistic variable value, the mass flow rate in the HVAC system will be the product of the output value and the total mass flow rate.

#### 2.2.4. Fuzzy Rules

The primary component of the fuzzy logic system was the fuzzy rules. It was crucial to define the fuzzy rules properly otherwise the objective of the controller would not be achieved. The fuzzy rules were designed with a primary function to regulate in-cabin PMV and CO_2_ levels within the pre-defined desirable ranges. Its secondary function was to achieve that without overspending energy on the blower and the cooling unit. To achieve that, a set of 140 rules were defined for the FLC. Some examples are listed as follows:

1. If PMV is Cool AND cabin temperature is Cold AND CO_2_ concentration is High, THEN blower velocity is High, AND Heating is turned ON AND recirculation flap opening is Large.
2. If PMV is Hot AND cabin temperature is Hot AND CO_2_ concentration is High, THEN blower velocity is High, AND Cooling is turned ON AND recirculation flap opening is Large.
3. If PMV is Comfort AND cabin temperature is Comfort AND CO_2_ concentration is Low, THEN blower velocity is OFF, AND HVAC is turned OFF AND recirculation flap opening is Small.

## 3. Simulation Setup

### 3.1. Input Parameters

The proposed fuzzy controller was implemented using the fuzzy logic toolbox in MATLAB Simulink. In this paper, summer temperature data were chosen for evaluating the proposed fuzzy controller, as it required the controller to trade off between its cooling outcome and energy consumption. In the simulation model, PMV, CO_2_, and cabin temperature were the input variables for the FLC. These input variables were specified as membership functions, where each element of the input was mapped to a value between 0 and 1 (see Appendix A for further explanation).

The outdoor temperature fluctuation was simulated as a sinusoidal function, with its max (40 °C), min (15 °C), and mean (27.5 °C) temperatures obtained from the actual data of Melbourne, Australia, in the summer of 2021 [33]. The lowest and highest temperatures recorded were 15.9 °C and 39.5 °C, respectively. An outdoor environment with a relative humidity of 50% was considered throughout the simulation period. The simulation was performed by assuming that the volume of the cabin is 3 m^3^, the airflow duct area is 0.0150 m^2^, and the mass flow rate of the refrigerant and coolant is 0.2 kg/s with a heat transfer rate of 6.5 kW and 4.8 kW, respectively [25,26]. Additionally, the refrigerant entering the evaporator is assumed as 4 °C, and the engine coolant entering the heater radiator is 90 °C throughout the simulation [25]. In the first stage, the velocity of air inside the cabin is the measure of blower air velocity. In addition to that, engine heat transfer and solar radiation through the windshield have been ignored in this simulation. The heat generated by the occupants, convection, and conductive heat transfer between in-cabin and outdoor environments has been considered. The simulation was performed by further assuming the average metabolic rate between 1.2 to 1.4 MET and the clothing insulation was assumed less than 1.5 clo (ASHRAE 55 Table 5.1 and 5.3 [2]). In addition, when the recirculation flap was closed, the vehicle cabin behaved as a sealed compartment where the leakage was negligible, such that no leakage occurred during the simulation except for the heat loss through conduction and convection.

### 3.2. Simulation Testing Conditions

The proposed FLC was designed to regulate the thermal comfort in terms of PMV and the cabin IAQ in terms of CO_2_ concentration as follows:

1. PMV index between −1 and 1;
2. Cabin temperature between 21 °C and 24 °C;
3. CO_2_ concentration below 650 ppm.

Two scenarios with different numbers of occupants (one and three) inside the vehicle cabin were simulated to investigate the effect of different numbers of occupants and to determine the adaptiveness of the proposed smart HVAC controller.

Furthermore, for comparison purposes, two other FLC controllers with recirculation flaps being open or closed at all times were introduced in the simulations to investigate the importance of incorporating CO_2_ concentration regulation in HVAC controller design.

### 3.3. Energy Consumption Measurement

In a conventional HVAC system, the design of the compressor parameters for controlling the refrigerant flow was driven by the HVAC load capacity. In the simulation, the energy consumption of the HVAC system was indicated using the average mass flow rate of the refrigerant in terms of kilogram per second (kg/s). The energy consumption for the heater was neglected as only waste heat from the engine was utilized during the summer season to heat the cabin air.

## 4. Results and Discussion

**Figure 5:**
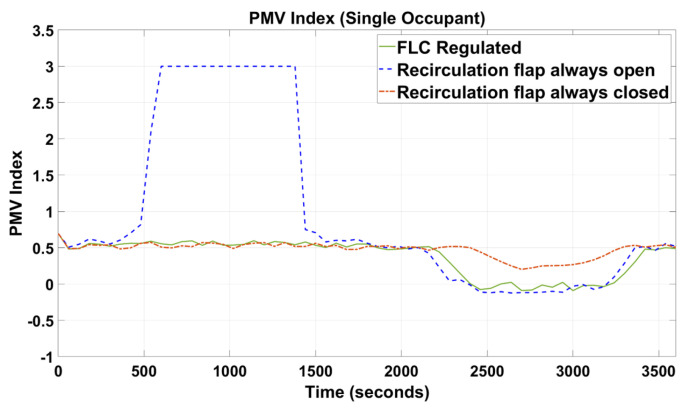
PMV index of the simulated vehicle cabin with one occupant.

**Figure 6:**
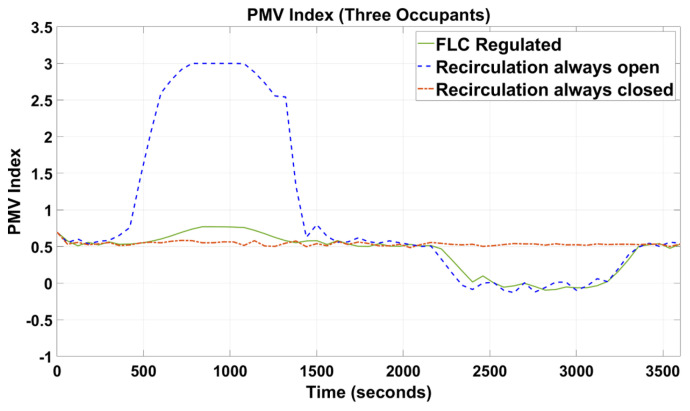
PMV index of the simulated vehicle cabin with three occupants.

**Figure 7:**
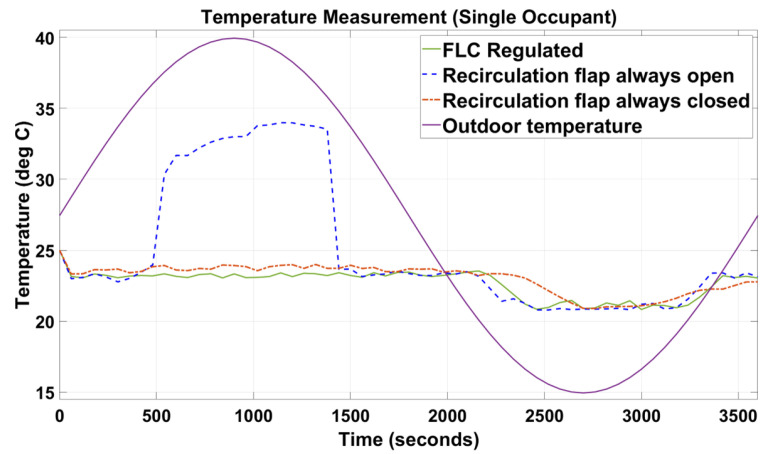
Indoor temperature of the simulated vehicle cabin with one occupant and outdoor temperature.

**Figure 8:**
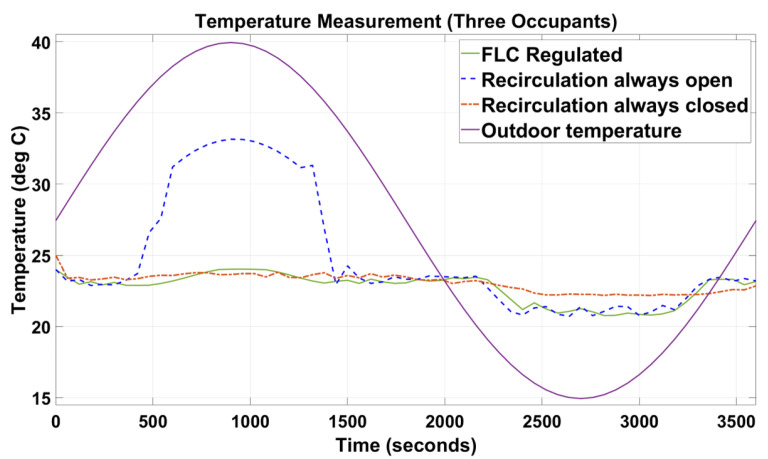
Indoor temperature of the simulated vehicle cabin with three occupants and outdoor temperature.

**Figure 9:**
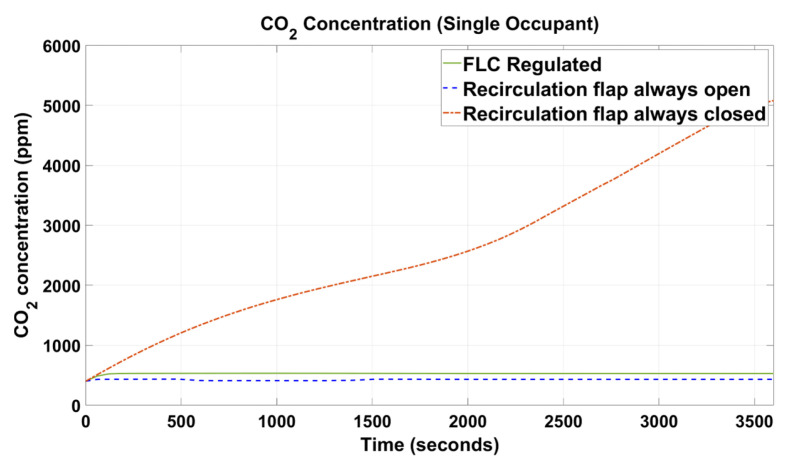
CO_2_ concentration of the simulated vehicle cabin with one occupant.

**Figure 10:**
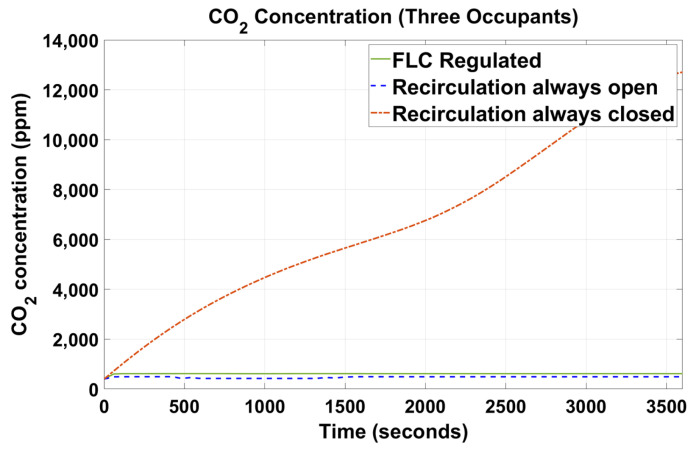
CO_2_ concentration of the simulated vehicle cabin with three occupants.

Figure 5, Figure 6, Figure 7, Figure 8, Figure 9 and Figure 10 show the simulation results of the HVAC controllers under test in regulating the thermal comfort of the occupants in terms of PMV, and the IAQ in terms of CO_2_ inside a simulated vehicle cabin with different numbers of virtual occupants and a time-varying outdoor temperature. The impacts of the number of occupants on PMV, CO_2_ concentration, energy consumption and performance indicators are presented next.

### 4.1. PMV Results

In Figure 5 and Figure 6, when the recirculation flap was forced to be opened during the simulation, it was observed that the PMV index started rising drastically at t = 300 s and reached the maximum threshold, i.e., PMV = 3. In Figure 7 and Figure 8, when the outdoor temperature started to decrease, the PMV index began to reduce at t = 1200 s.

For the other two simulation setup conditions, i.e., when the recirculation flap was either forced to be closed or the recirculation flap controlled by the proposed FLC, the PMV index was observed to be regulated between the predefined threshold values between −1 and +1.

Since cabin temperature was a key parameter of the PMV index, as shown in Figure 6 and Figure 9, when the recirculation flap was forced to be always open, the indoor cabin temperature waveform shadows the outdoor temperature. Again, in Figure 6 and Figure 9, under the FLC-regulated and recirculation flap closed conditions, their in-cabin temperature was regulated between 21 and 24 °C as intended.

When designing intelligent HVAC system controllers, previous studies [18,19,20,21] have only considered cabin temperature as the primary factor to measure thermal comfort and cabin IAQ. In this paper, cabin temperature, relative humidity, and air velocity were considered as variable input factors to predict the PMV index based on the simulated outdoor environmental parameters. At the same time, human metabolic rate and clothing insulation were designed to change manually during the simulation. The results (Figure 5 and Figure 6) demonstrated that our novel approach was successful in maintaining the thermal comfort range by regulating the PMV index between −1 and +1.

### 4.2. CO_2_ Concentration

Figure 9 and Figure 10 show the CO_2_ concentration results of the proposed fuzzy HVAC controller versus its counterparts with one virtual occupant and three virtual occupants, respectively.

As expected, for the setup with the recirculation flap forced to be always closed, the CO_2_ concentration kept increasing monotonically throughout the simulation period, while for the other two setups, the CO_2_ concentration was regulated below the predefined maximum threshold of 650 ppm. It was also observed that the proposed FLC could regulate the in-cabin CO_2_ concentration close to the setup with the recirculation flap forced to be always open. From Figure 9, when with one occupant presence, CO_2_ concentration was regulated at 430 ppm when the recirculation flap was forced open and at 528 ppm in the system controlled by the proposed FLC. Similar results were observed in Figure 10 when three occupants were present. The FLC regulated with the recirculation flap was forced to be open, and the CO_2_ concentration was regulated at 490 ppm and 615 ppm for setups with one and three occupants, respectively.

Studies by Russi et al. [8,9] traded off energy efficiency for regulating the CO_2_ concentration to maintain better cabin IAQ. We developed a fuzzy logic-controlled HVAC system to address the issue. Table 5 demonstrated that the occupant’s thermal comfort and cabin IAQ could be regulated with the proposed FLC without causing a significant increase in the energy consumption indicator, which was estimated using the refrigerant mass flow rate (kg/s). The results (Table 5) further showed that the mass flow rate values were ½ times higher when compared with the recirculation flap closed condition and 9/10 times lower when compared with the recirculation flap open.

As shown in Figure 5, Figure 6, Figure 7, Figure 8, Figure 9 and Figure 10, the proposed FLC, which was designed and developed using the fuzzy logic toolbox in Simulink, has successfully regulated the PMV index and CO_2_ concentration within their predefined desirable thresholds.

In the simulation setup with the recirculation flap always closed and with one occupant, at t = 2450 s (Figure 5), the PMV index reduced relatively slower than the other two setups (i.e., FLC-regulated and recirculation flap always closed) when the external temperature started to drop. This was due to the in-cabin air continuing to recirculate regardless of the changes in the outdoor temperature. Therefore, the only possible way for the in-cabin air to be cooled down was through convective and conductive heat transfer, which was a relatively slow process. As such, when t was between 2450 s and 3350 s, even when the outdoor temperature increased rapidly from 15 to 20 °C, the in-cabin temperature reacted slowly as the vehicle compartment was assumed to be perfectly sealed. In contrast, for the other two setups, outdoor air was allowed to flow in, and as the outdoor air at that instance was relatively cold (15 °C to 20 °C), that cooled down the in-cabin temperature relatively quickly. As a result, their PMV values could be regulated around 0 during that simulated period. Furthermore, in Figure 7, during that simulated period, when the in-cabin temperature went below 21 °C, the blend door was opened to allow some amount of air to pass through the HVAC radiator to increase the temperature such that the in-cabin temperature was regulated at the desirable level, which explained why the in-cabin temperature and, thus, the PMV index, did not reduce further. When the recirculation flap was always closed and with three occupants (Figure 6), the PMV index was regulated at 0.5 with negligible fluctuations of +/−0.1. Even though the cabin was cooled down due to convective and conductive heat transfers, such a process was slow. Nevertheless, there was extra heat generated by the two new additional occupants, which caused the in-cabin temperature and, thus, the PMV index, to increase.

In a conventional vehicle cabin, even when the cabin is assumed to be completely sealed, there will be leakage from the indoor to an outdoor environment. In the simulated results, the PMV index was analyzed with +/- 0.1 tolerance. In the future, during experimental validation, the tolerance for the PMV index can be determined using standard deviation.

When the recirculation flap was closed, there was no alternative channel for the in-cabin air to escape and the CO_2_ concentration started to accumulate and reach an alarming level (Figure 9 and Figure 10). The increase in CO_2_ concentration was due to the presence of occupants who generated CO_2_ at a rate of 0.01 g/s per person. In reality, the vehicle cabin is not perfectly sealed, and such exceeding high values as shown in the results should not be realistic. However, with the recirculation flap closed, the in-cabin CO_2_ concentration could still significantly increase to a level that could cause drowsiness or other health hazards to the occupants. Hence, to avoid such circumstances, CO_2_ concentration should be monitored and regulated below a desirable threshold of 650 ppm. The proposed FLC was able to regulate the in-cabin CO_2_ concentration below such a limit. Moreover, the proposed FLC could regulate the CO_2_ concentration closely as that in the setup with the recirculation flap always open. The results demonstrated that the proposed FLC was effective in regulating the in-cabin CO_2_ concentration by adaptively controlling the opening and closing of the recirculation flap according to in-cabin temperature and CO_2_ readings.

### 4.3. Energy Consumption Measurement

In the first stage, the average area under the curve of the product of the HVAC system on-time (1-On, 0-Off) and its corresponding refrigerant mass flow rate (% of the max mass flow rate) was used as an indicator to estimate the HVAC system energy consumption. In conventional vehicles, the energy consumption will be measured based on the power utilized by the blower fan, compressor, and electric heater. The energy consumption was evaluated under the influence of different extrinsic factors and different controllers in different test conditions. The evaluation was used to compare the energy efficiency and the effectiveness of the proposed FLC versus its counterparts in regulating PMV and CO_2_ levels.

The corresponding average area under the curve (in kg/s) is tabulated in Table 5. It was observed that when the recirculation flap was forced to be always open, its corresponding indicator could be higher than those in setups with the proposed FLC and the recirculation flap always closed, by an order of magnitude. In contrast, the refrigerant mass flow rates for the system with the proposed FLC and recirculation flap always closed were lower, thus showing that energy consumption could be lower if the recirculation flap was partially or completely closed.

### 4.4. Performance Indicators

Ideally, an HVAC controller should aim to regulate PMV within the comfort range around PMV = 0 and to keep CO_2_ concentration lower than 650 ppm. Therefore, in this paper, the root mean square error (RMSE) was used to evaluate the performance of the HVAC controllers in achieving the comfort range, whereas the mean average error (MAE) was used for quantifying their CO_2_ regulating performances. The RMSE (Table 6) measured the deviation of the PMV index from the absolute thermal comfort (i.e., PMV index = 0) under various testing setups and conditions. The MAE (Table 7) measured the deviation of the CO_2_ concentration above the baseline concentration presented in the outdoor environment air (i.e., 400 ppm). From the results, it was observed that the proposed FLC has a similar performance in regulating PMV as that of the setup with the recirculation flap always closed. The RMSE value was almost 1/3 of that of the setup with the recirculation flat always open. Regarding the MAE for in-cabin CO_2_, as expected, the setup with the recirculation flap always open yielded the lowest value. While the proposed FLC was five times higher, nonetheless, its corresponding ppm value was still below the target threshold of 650 ppm. In contrast, the setup with the recirculation flap always closed has an exceedingly high value of MAE for CO_2_.

In Table 8, the proposed FLC was compared with other controller setups in regulating thermal comfort and in-cabin IAQ, together with their energy consumption indicators. To ease comparisons, each controller was assessed using a star rating system. The rating was intended to visualize the relative performances of the controllers in different aspects. It can be seen that when the recirculation flap was forced to be closed at all times, the PMV index and cabin temperature were regulated within the occupant’s thermal comfort threshold ranges. In return, the cabin IAQ degraded in terms of CO_2_ concentration as it started accumulating above the occupant’s safety threshold limit. When the recirculation flap was forced to be always open, a better cabin IAQ was achieved in terms of CO_2_ concentration. As shown in Table 8, the proposed FLC showed significantly improved results in regulating thermal comfort and cabin IAQ with average energy consumption in terms of mass flow rate when compared with the other two setups that lacked control of air recirculation. This indicated the importance of incorporating CO_2_ concentration in the controller design process.

From the energy consumption indicator, it was observed that the proposed FLC could achieve a reasonable tradeoff between regulating PMV and CO_2_ concentration while maintaining an efficient level of energy consumption. Although the energy consumption indicator was higher than that of the setup with the recirculation flap closed, it could still regulate the in-cabin CO_2_ concentrations at the desirable level, which its counterpart (recirculation flap always closed) failed to do. When compared to the setup with the recirculation flap open, the proposed FLC only consumed 1/5 of the total energy (in terms of the indicator) and it was able to regulate the PMV and CO_2_ within the predefined desirable levels.

## 5. Future Work

This was preliminary work to study the importance of incorporating CO_2_ concentration and PMV in the design of vehicle HVAC controllers. FLC mechanisms were adopted due to their simplicity and effectiveness in handling problems with a relatively small number of nonlinear variables. Future work will be focused on testing the proposed FLC for other weather condition profiles and evaluating the energy consumption for HVAC systems with different architectures. Further, the proposed FLC would be experimentally validated using sensor technology to capture the relative humidity, cabin temperature, cabin velocity, and CO_2_ concentration. The measured values will be given as input to the FLC and the outcome will be analyzed. Regarding the controller, machine-learning approaches will be adopted for fine-tuning the fuzzy logic membership functions automatically and adaptively according to different room settings and environmental parameters. Reinforcement learning will be incorporated with a fuzzy logic system to fine-tune the membership functions and fuzzy rules for regulating the PMV index and other IAQ parameters to be within the preferred levels in an energy-efficient manner.

## 6. Conclusions

The objective of this paper was to develop an intelligent controller to regulate the thermal comfort and IAQ of a vehicle compartment without significantly increasing the corresponding energy consumption. As discussed earlier, conventional vehicle HVAC systems are the primary energy consumers in vehicles. Here, the FLC-controlled HVAC system performed well in terms of regulating thermal comfort and IAQ with average energy consumption. This helps to increase the driving efficiency of the vehicles. Thermal comfort and IAQ were measured in terms of in-cabin PMV and CO_2_ concentration, respectively. The control mechanism was realized with an FLC, which regulated both PMV and CO_2_ concentration by adjusting the control parameters of the vehicle HVAC system. The performance of the proposed vehicle HVAC controller was evaluated using computer simulations via MATLAB Simulink and compared with two other setups that lacked control of air recirculation. Simulation results showed that the proposed controller was capable of regulating both PMV and CO_2_ concentration within all predefined desirable threshold limits at the expense of only a small increase in energy consumption. In contrast, systems controlled by their counterparts have violated some of the thresholds.

## Figures and Tables

**Table 1:** Parameters of thermal comfort and IAQ incorporated in the studies of vehicle HVAC system controller designs over the past five years.

Literature, Year	Controlling Mechanisms/Software Used	Parameters Considered while Measuring Thermal Comfort and IAQ	Limitations
Wei et al., 2022 [5]	GT-SUITE	CO_2_, PM, and VOCs	While controlling CO_2_, recirculation flap degrees were not discussed.
Russi et al., 2022 [9]	Model predictive control	PM and VOCs	Without considering in-cabin CO_2_ concentration, their results indicated that a higher recirculation level can lower in-cabin PM levels and energy consumption.
He et al., 2022 [18]	Graph Convolutional Network, Gated Recurrent Unit	Cabin temperature	Use only the cabin temperature to estimate the occupant’s thermal comfort.
Sagoian et al., 2021 [19]	Ensemble—a combination of linear regression and random forest algorithm	Cabin temperature	Use only the cabin temperature to predict the occupant’s thermal comfort and estimate energy consumption.
Russi et al., 2021 [8]	Arduino based sensors system	PM and VOCs	Without considering in-cabin CO_2_ concentration, their results indicated that a higher recirculation level can lower in-cabin PM levels and energy consumption.
Ramsey et al., 2021 [20]	Energetic macroscopic representation, MATLAB	Cabin temperature and relative humidity	Use only the cabin temperature and relative humidity to estimate the occupant’s thermal comfort.
Lim et al., 2021 [21]	Artificial neural network	Cabin temperature	Aimed for trading-off heating for battery lifetime. Use only the cabin temperature to estimate the occupant’s thermal comfort.
Xie et al., 2020 [13]	Dynamic programming	Cabin temperature and air velocity	Estimated PMV’s accuracy degraded under extreme temperature scenarios
Warey et al., 2020 [22]	Artificial neural network, linear regression, and random forest algorithm	Cabin temperature, relative humidity, and air velocity	It uses three machine learning algorithms. Even though cabin temperature, relative humidity and air velocity were considered to estimate occupants’ thermal comfort, only cabin temperature was considered while training all three machine learning algorithms.
Schaut et al., 2020 [23]	Model predictive control	Cabin temperature, relative humidity, and air velocity	Energy minimization was achieved via an optimum on–off schedule of the evaporator.
Lahlou et al., 2018 [16]	Dynamic programming	Cabin temperature and relative humidity	Thermal comfort was achieved with an increase in energy consumption. CO_2_ was not considered.

**Table 2:** Fuzzy logic membership range that was used as input and output variables.

Reference		Variable	Range
ASHRAE Standard 55, 2020 [2]; Bretones et al., 2015 [30]	Input	PMV	−3 to +3
		CO_2_ concentration (PPM)	0 to 1500
		Vehicle cabin temperature (°C)	0 to 50
Sivakumar, M., 2022 [25]	Output	Blower velocity (m/s)	0 to 1
		HVAC ON/OFF	0 to 1
		Mass flow rate of refrigerant and coolant	0 to 1
		Recirculation flap	0 to 1

**Table 3:** Fuzzy controller input linguistic values that are used by the membership function.

Reference	Input Variable	Linguistic Variable	Values
ASHRAE Standard 55, 2020 [2]	PMV	Cold	−3 to −2
		Cool	−2 to −1
		Slightly Cool	−1 to −0.5
		Neutral	−0.5 to +0.5
		Slightly Warm	+0.5 to +1
		Warm	+1 to +2
		Hot	+2 to +3
Bretones et al., 2015 [30]	CO_2_ (ppm)	High	>1000
		Medium	600−1000
		Moderate	400−600
		Low	≤400
ASHRAE Standard 55, 2020 [2]	Temperature (°C)	Cold	0–18
		Cool	18–21
		Comfort	21–24
		Warm	24–27
		Hot	27–50

**Table 4:** Fuzzy controller input linguistic values for membership functions.

Output Variable	Linguistic Variable	Values
Blower velocity	OFF	0–0.1
	Low	0.1–0.35
	Medium	0.35–0.65
	High	0.65–0.85
	Maximum	0.85–1
HVAC	Cooling	0–0.45
	OFF	0.45–0.55
	Heating	0.55 −1
Mass flow rate of refrigerant and coolant	OFF	0–0.1
	Low	0.1–0.35
	Medium	0.35–0.65
	High	0.65–0.85
	Maximum	0.85 −1
Recirculation flap	Low	0–0.25
	Moderate	0.25–0.50
	Medium	0.50–0.75
	High	0.75–1

**Table 5:** Average area under the curve of the product of the HVAC on time and its corresponding mass flow rate (kg/s) in simulations with different setups.

Description	One Occupant	Three Occupants
FLC regualted	0.067	0.114
FLC with recirculation flap always open	0.284	0.297
FLC with recirculation flap always closed	0.031	0.045

**Table 6:** Root mean square error for the PMV index with different simulation setups.

Description	One Occupant	Three Occupants
FLC-regulated	0.445	0.5
FLC with recirculation flap always open	1.406	1.451
FLC with recirculation flap always closed	0.485	0.533

**Table 7:** Mean Average Error for CO_2_ (ppm) in simulations with different setups.

Description	One Occupant	Three Occupants
FLC-regulated	127.829	214.028
FLC with recirculation flap always open	25.377	72.395
FLC with recirculation flap always closed	2230.120	6286.023

**Table 8:** An overview of the performance indicators of the vehicle HVAC controllers under test.

	PMV Index	Cabin Temperature	CO_2_ Concentration	Energy Consumption
FLC-regulated	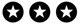	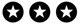	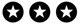	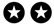
FLC with recirculation flap always open	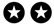	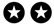	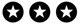	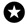
FLC with recirculation flap always closed	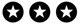	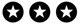	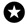	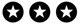

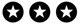
—Good,
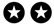
—Average,
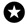
—Poor.

**Figure A1:**
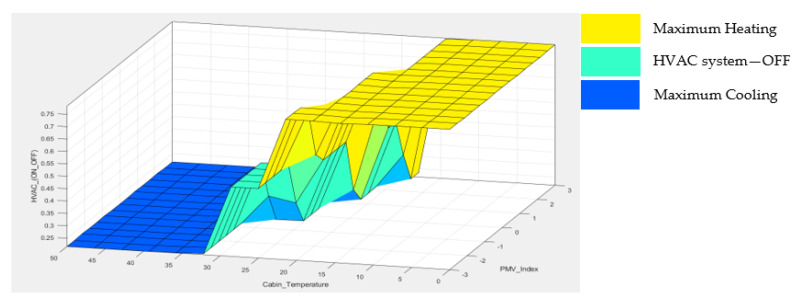
Mapping of the PMV index and cabin temperature membership function to control the HVAC system on/off and mass flow rate.

**Figure A2:**
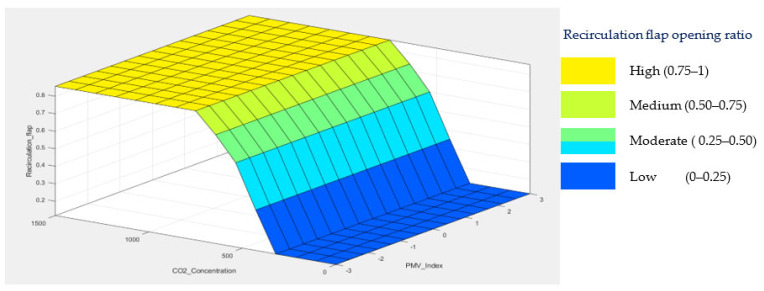
Fuzzy logic membership function of the PMV index and CO_2_ concentration designed to regulate the HVAC system recirculation flap.

## Data Availability

Not applicable.

## Abbreviations

ASHRAE	American Society of Heating, Refrigeration, and Air-Conditioning Engineers
EN	European Standards
FLC	Fuzzy Logic Controller
HVAC	Heating Ventilation and Air-Conditioning
IAQ	Indoor Air Quality
ISO	International Organization for Standardization
PI	Proportional Integrate
PID	Proportional Integrate Derivative
PM	Particulate Matter
PMV	Predicted Mean Vote
TEV	Thermostatic Expansion Valve
trapmf	Trapezoidal Membership Function
trimf	Triangular Membership Function
VOC	Volatile Organic Compounds

## Appendix A

This section explains the construction of membership functions in the proposed FLC.

In Figure A1 and Figure A2, the *x* and *y*-axes represent the input variables, and the *z*-axis represents the output variables in the FLC. Figure A1 illustrates the mapping of the PMV index and cabin temperature to control the on/off and mass flow rate of the refrigerant and coolant of a vehicle HVAC system obtained from those 140 fuzzy rules. As per the fuzzy rules designed, when the cabin temperature was less than 18 °C or greater than 30 °C, the outputs from the FLC notified the HVAC system to deliver the maximum ratio of refrigerant or hot coolant to regulate the thermal comfort within the pre-defined level. At the same time, when the cabin temperature fluctuated between 18 °C and 30 °C, the FLC reduced the mass flow rate (Table 4) accordingly.

Figure A2 illustrates the mapping of the PMV index and CO_2_ concentration to control the recirculation flap of the vehicle HVAC system through fuzzy rules. As described in Figure A2, based on the CO_2_ concentration fluctuations, the recirculation flap will be opened or closed accordingly. When the CO_2_ concentration inside the vehicle cabin reached 650 ppm, the recirculation flap opened (High (0.75–1)) to allow more fresh air flow into the vehicle cabin. As such, a better cabin IAQ can be maintained in terms of CO_2_ concentration.
